# Gaining insights in the nutritional metabolism of amphibians: analyzing body nutrient profiles of the African clawed frog, *Xenopus laevis*

**DOI:** 10.7717/peerj.7365

**Published:** 2019-08-07

**Authors:** Andrea Brenes-Soto, Ellen S. Dierenfeld, Guido Bosch, Wouter H. Hendriks, Geert P.J. Janssens

**Affiliations:** 1 Laboratory of Animal Nutrition, Faculty of Veterinary Medicine, Ghent University, Merelbeke, Belgium; 2 Animal Science Department, University of Costa Rica, Ciudad Universitaria Rodrigo Facio, San Jose, Costa Rica; 3 Ellen S. Dierenfeld LLC, Saint Louis, MO, United States of America; 4 Animal Nutrition Group, Wageningen University, Wageningen, The Netherlands

**Keywords:** Metabolism, Nutrition, *Xenopus laevis*, Body composition, Amino acids, Fatty acids

## Abstract

Whole bodies of *Xenopus laevis* (*n* = 19) were analysed for chemical composition and morphometrics. The nutrient profile (macronutrients, amino acids, fatty acids and minerals) was evaluated by sex; interactions among variables with body weights and lengths, and comparisons made with different species of marine and fresh water fish. Significant differences were found in morphometric measurements, water content, several minerals and fatty acids between sexes of *X. laevis*. Amino acid profiles differed in methionine, proline and cysteine, which could underlie different metabolic pathways in frogs when compared to fish. In addition, fatty acid profiles revealed more monounsaturated and *n* − 6 polyunsaturated fatty acids in frogs than in fish, more similar to values reported for terrestrial than aquatic vertebrates. Important interactions were also found between body measurements and fat, calcium, and phosphorus, as well as between essential and non-essential amino acids. The results indicate that frogs might have particular biochemical pathways for several nutrients, dependent on sex and linked to body weight, which ultimately could reflect specific nutrient needs.

## Introduction

The nutritional requirements of species are fundamental to formulate adequate diets for captive individuals. In the case of amphibians, there is a dearth of information regarding nutrition, where research has been hampered by species’ physiological adaptations and their ectothermic nature, which make them especially sensitive to changes in environmental conditions (temperature, humidity, seasonality, photoperiodicity, rainfall, etc.) (Duellman & Trueb, 1994; Carmona-Osalde et al., 1996; Ferrie et al., 2014). Since worldwide recognition of the widespread decline of many amphibian species (Bishop et al., 2012; Whittaker et al., 2013), specialists and conservation organizations urge for more captive programs to be developed to prevent potential population extinction (Schad, 2007), and adequate nutrition is essential for the health and reproductive success of captive amphibians (Ferrie et al., 2014).

Anurans present a challenge for the establishment of dietary nutrient recommendations, with variations depending on the life stage, habitat and physiology (Carmona-Osalde et al., 1996; Browne, 2009; Ferrie et al., 2014). For instance, fluid and electrolyte homeostasis in frogs is maintained by the balance between the activity of the kidneys, urinary bladder and skin, the latter having high permeability to contribute to osmoregulation and fluid homeostasis (Campbell et al., 2012). Likewise, nitrogen metabolism and excretion pathways are unique in the case of amphibians (Duellman & Trueb, 1994) and these processes can directly affect amino acid requirements (Kaneko, Harvey & Bruss, 2008; Ferrie et al., 2014). Usually, the provision of adequate amino acids is met only through consideration of the essential amino acids. However, the concept of “functional” amino acids encompasses a wider range of processes, taking into account that these substances play multiple roles in animal physiology, whether essential or not (Wu et al., 2013). Some non-essential amino acids like arginine, glutamine, glutamate, glycine, proline, cysteine and taurine, have been shown to participate actively in other functions such as gas synthesis to enhance blood flow, nutrient transport and protein deposition; enhancement of immune function has also been demonstrated across species (Wu et al., 2013). Therefore, multiple biochemical pathways must thus be considered in ascertaining nutrient requirements.

Aside from protein constituents, energetics involves a series of metabolic characteristics very particular in amphibians. There is much variation in energy production and expenditure (both aerobic and anaerobic) during resting and activity, as well as through natural behaviours like foraging, courtship, fighting and vocal communication. Consequently, amphibians can use multiple substrates to support metabolic activities, and seasonal cycles of energy storage and utilisation (Wells, 2007). Although energy may be stored as glycogen, proteins or lipids, amphibians preferentially utilise the latter. Triglycerides, as main energy reserves, are stored in the abdominal fat bodies and depots in the rest of the body, for production of oocytes, gametes, metabolic support during dormancy, and gonadal maintenance (Fitzpatrick, 1976). Reproduction may thus depend on whole body fat stores rather than on fat bodies alone (Pinder, Storey & Ultsch, 1992). Additionally in frogs, fatty acids play a key role in cell membrane function by changing membrane composition, as an ectothermic adaptive response during temperature acclimation (Pinder, Storey & Ultsch, 1992). In this regard, authors reported changes in the proportions of unsaturated and polyunsaturated fatty acids in the neutral lipid and phospholipid fractions of liver in the edible frog (*Rana esculenta*) (Baranska & Wlodawer, 1969), as well as in the lipid matrix of microsomal and mitochondrial membranes from skin of the common frog (*Rana temporaria*) (Lagerspetz & Laine, 1984).

Certainly, one method to estimate nutritional requirements is the use of digestibility and balance studies (Carmona-Osalde et al., 1996; McDonald et al., 2011; Silva de Castro et al., 2012). Nonetheless, the validity of these studies could be limited, unless requirements for fractional turnover, excretion in faeces or synthesis of other substances not arising directly from food, as well as over/underestimation of energy expenditures, are considered (McDonald et al., 2011). In this regard, some amphibians have shown a high digestive flexibility, where changes in diet, frequency of food intake and seasonality may influence the enzymatic activity as well as the morphology of the digestive tract (Secor, 2001; Sabat, Riveros & López-Pinto, 2005; Naya, Bozinovic & Sabat, 2008).

The body’s nutrient profile can also yield valuable information on what is required by an animal, considering distribution and associations in the whole body as a reflection of the animals metabolic activity and dynamics. Interesting findings in this regard have been reported in several species such as the arctic caribou (Gerhart et al., 1996), the laboratory rat (Donato et al., 2006) and several avian species (Daan, Masman & Groenewold, 1990), where body composition (in terms of macronutrients) and energy turnover varied with physiological state, seasonal changes and diet. Moreover, body composition can also be impacted by factors including sex and size (Szendro et al., 1998; Hall et al., 2007). Overall, few detailed studies examining diet and nutritional biochemistry with amphibian species exist (Olvera-Novoa, Ontiveros-Escutia & Flores-Nava, 2007; Ferrie et al., 2014) and, therefore, nutritional recommendations are often based on other species models. A straightforward analysis of the nutrient concentrations in amphibian bodies cannot deliver clear nutritional guidelines, but it can provide relationships among nutrients that can—as a first step—provide insights into amphibian nutrient metabolism.

Regardless of conservation programs, anurans are maintained in captivity as laboratory animals as well as part of collections in zoos and herpetaria (Browne, 2009; Brown & Rosati, 1997). Likewise, they have been used as whole prey items of various captive feeding programs in zoos (Kwiecinski et al., 2006; Rosin & Kwiecinski, 2011) for certain predators (e.g., storks, snakes, mammals, other anurans), frequently and/or seasonally (Schaaf & Garton, 1970; Hoyo, Elliot & Sargatal, 1992; Schairer, Dierenfeld & Fitzpatrick, 1998; Pearl & Hayes, 2009). In this respect, the African clawed frog, *Xenopus laevis*, has a long history in scientific research, as a major non-mammalian laboratory animal model in vertebrate physiology, biochemistry and cellular biology, and other scientific fields (Green, 2010). We use this common captive-bred frog species *X. laevis*, as a model to investigate body composition as a first indication of nutrient dynamics and metabolism, and evaluate effects of sex and body size.

## Materials and Methods

### Animals and housing

Nineteen adult *X. laevis* (*n* = 9 females, *n* = 10 males) from a healthy colony were used. Animals were housed in groups of three or four animals, separated by sex, in six 65 L tanks (60 ×30 ×36 cm) darkened on three sides, and provided with PVC pipes as hiding sites. Average water temperature of the tanks was controlled by a heater (Juwel^®^ 50 W, Juwel Aquarium AG & Co, Rotemburg, Germany) and maintained at 22 °C with the photoperiod set at 12:12 h light:dark. Water quality of the tanks was monitored every two weeks and kept under the following conditions: 0.01–0.05 mg L^−1^ nitrites, 0.5–10 mg L^−1^ nitrates (JBL^®^ GmbH & Co., Germany); <0.01 mg/L ammonia and 0.05 mg L^−1^ ammonium (Colombo^®^, The Netherlands). Water hardness was maintained at 359 mg L^−1^, and pH at 7.6. A UV bulb (Exo Terra^®^ 11W, Rolf C. Hagen Inc., Montreal QC, Canada) was placed at five cm from the water surface twice a week in each tank, for an eight-hour exposure period. Weights (W) and lengths (L) of the frogs were measured monthly using a digital scale (OHaus CS Series ± 1 g) and a Vernier calliper (±0.1 mm), respectively. The experiment was carried out following the guidelines of the EU Directive 2010/63/EU for animal experiments, and approved by the Ethical Committee of the Faculty of Veterinary Medicine and the Faculty of Bioscience Engineering of Ghent University, No. EC 2015/133.

### Diet and feeding

During the six month trials, animals were fed three times per week a pelleted diet made in the Laboratory of Animal Nutrition, composed of shrimp meal (66.0%), soy bean meal (30.25%), calcium phosphate (0.75%), beef fat (1%), multivitamin supplement (0.50%) and rice syrup (1.5%). The composition of this diet was based on studies performed in bullfrogs, *Rana* (*Lithobates*) *catesbeiana* (Silva et al., 2014; Mansano et al., 2016; Zhang et al., 2015). The nutrient composition of the diet is presented in Table 1. The weekly food intake (dry matter) per tank was 5.2 ± 0.6 g for females and 2.5 ± 0.4 g for males.

**Table 1:** Analysed chemical composition of the diet (dry matter basis) fed to *Xenopus laevis*.

Proximates (%)	Amount
Dry matter^a^	95.1
Crude protein	39.9
Crude fat	11.6
Ash	19.3
Macro minerals (g/kg)	
Calcium	43.2
Magnesium	2.4
Phosphorus	9.7
Potassium	11.1
Sodium	4.0
Micro minerals (mg/kg)	
Copper	39.5
Iron	196.5
Manganese	30.8
Selenium	0.5
Zinc	62.2
Amino acids (%)	
Alanine	6.8
Arginine	7.1
Aspartic acid	11.6
Cysteine	1.4
Glutamic acid	16.2
Glycine	5.5
Histidine	2.8
Isoleucine	5.0
Leucine	8.4
Lysine	6.3
Methionine	2.2
Phenylalanine	5.2
Proline	5.2
Serine	5.6
Threonine	5.0
Valine	5.7

**Notes.**

a As is basis.

### Chemical analysis

Euthanasia was performed through submersion of the frog in a solution of tricaine methanesulfonate (Green, 2010); the frogs were frozen, freeze-dried and ground to pass a one mm screen prior to analysis. Both whole animals and feed were analysed for proximate components (AOAC, 1984), amino acid content after defatting (Van Rooijen et al., 2014), and mineral profiles (Dermauw et al., 2013). Fatty acid profiles (Vlaeminck, Braeckman & Fievez, 2014) were conducted only on the frog samples.

All data from body composition (macronutrients, amino acids and fatty acids expressed as percentages of the sum of total) were further compared to other frog species (*Rana esculenta*) as well as marine and fresh water fish (Black Sea anchovy *Engraulis encrasicholus*, Commersons anchovy *Stolephorus commersonii*, cod *Gadus morhua* and tilapia *Oreochromis niloticus*). The choice was based on vertebrate species with similar habitat (aquatic), for which detailed whole body composition was available. This search was accomplished through a literature review using Google Scholar with a search term for whole body chemical composition of the selected species.

### Statistical analysis

All data were expressed as means and standard deviations, and were normally distributed. Analyses performed included univariate ANOVA to determine differences of morphometric measurements and nutrient profiles (denoted as concentrations on a dry matter basis) between sexes with significance declared at *p* < 0.05. All data were further combined to define relationships among morphometric measurements (weight and length) and nutrient levels through a multivariate analysis using principal components analysis (PCA), deemed relevant with a value above 0.5 and below -0.5 in the magnitude of each component. This analysis included the scores, as well as relative (percentages of the total) and absolute (concentrations per unit of dry matter) values of each nutrient, in order to identify associations independent of the dry matter (in the first case) as well as distribution in the tissues (in the second case).

The same analysis was performed with the residuals of all variables obtained after correcting the data for sex, to determine whether variations independent of sex could be detected. From each component, groups relevant for nutritional physiology were highlighted in clouds in the plots. All statistical analyses were conducted using the SPSS 23 program.

## Results

### Differences between sexes

**Figure 1:**
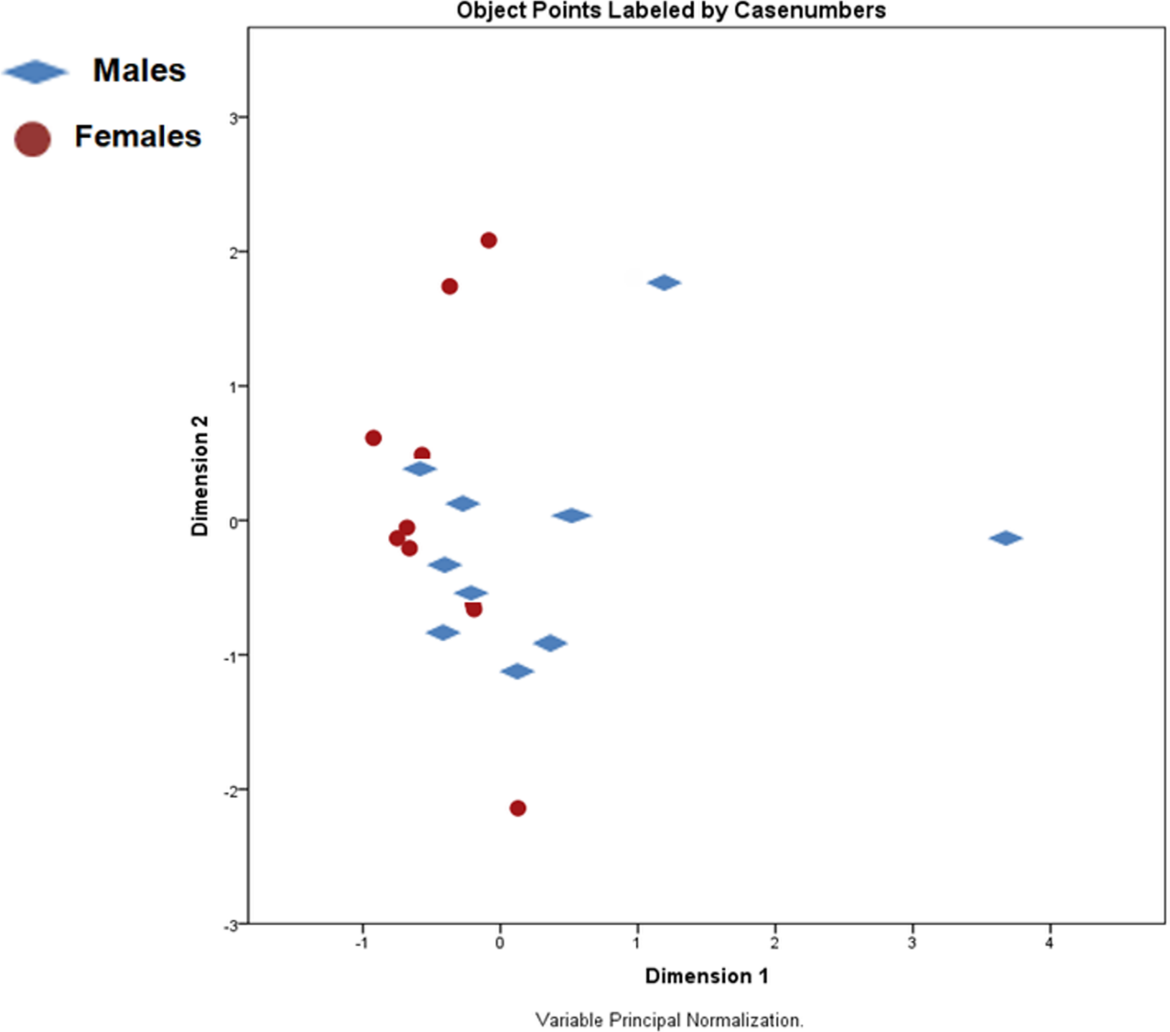
Principal components analysis (PCA) scores plot of male and female *X. laevis*.

The score plot (Fig. 1) shows no distinct separation between the two sexes. Yet, some parameters do appear linked to sex and size. Weights and lengths of females and males significantly varied (*p* < 0.001), averaging 83.2 ± 18.4 and 39.0 ± 6.6 g, and 79.0 ± 8.2 and 58.8 ± 3.9 mm, respectively. Besides these morphometric differences, females had a higher content of dry matter and less total ash than males, while crude fat and crude protein content did not differ. Calcium and phosphorus values were higher, and iron and selenium were lower in males compared to females (*p* < 0.05) (Table 2). Amino acid values are presented in Table 3. Although not significant, males presented numerically higher values than females. On the other hand, the fatty acid profiles exhibited by females had higher values of both saturated (SFA) and monounsaturated fatty acids (MUFA) (*p* < 0.05), while males had numerically more polyunsaturated fatty acids (both *n* − 3 and *n* − 6 PUFA) (Table 4).

**Table 2:** Mean+SD^a^ proximates and mineral composition (dry matter basis) of adult whole African clawed frogs (*Xenopus laevis*).

Component	Females *n* = 9	Males *n* = 10	*P*
Proximates (%)			
Dry matter	22.3 ± 3.5	19.9 ± 2.3	0.030*
Crude protein	53.2 ± 3.4	52.8 ± 2.4	0.807
Crude fat	20.1 ± 4.5	17.3 ± 3.6	0.143
Ash	12.5 ± 2.0	16.2 ± 2.3	0.020^*^
Macro minerals (g/kg)	****	****	
Calcium	45.7 ± 9.2	62.0 ± 10.5	0.020^*^
Phosphorus	28.4 ± 4.9	34.4 ± 5.1	0.019^*^
Magnesium	1.4 ± 0.1	1.5 ± 0.1	0.074
Potassium	8.6 ± 0.4	8.3 ± 0.9	0.385
Sodium	6.0 ± 1.2	6.1 ± 1.6	0.892
Micro minerals (mg/kg)	****	****	
Copper	11.7 ± 4.8	7.7 ± 3.6	0.072
Iron	183.7 ± 22.8	271.0 ± 48.7	<0.001^*^
Manganese	13.6 ± 4.4	14.6 ± 5.5	0.682
Selenium	1.19 ± 0.1	0.9 ± 0.1	<0.001^*^
Zinc	104.8 ± 19.6	108.4 ± 9.9	0.608

**Notes.**

a Standard deviation.

* *p* < 0.05.

**Table 3:** Mean+SD^a^ amino acid profiles of adult whole African clawed frogs (*Xenopus laevis*).

Amino acid	Females *n* = 9	Males *n* = 10	*P*	Females *n* = 9	Males *n* = 10	*P*	Pattern^b^
	(g/kg Dry matter)		**	(g/kg Crude protein)			
Alanine	34.4 ± 2.5	36.0 ± 3.0	0.234	64.7 ± 0.5	68.2 ± 0.6	0.164	84
Arginine	28.9 ± 2.6	29.9 ± 2.8	0.431	54.3 ± 0.5	56.6 ± 0.5	0.340	70
Aspartic acid^c^	50.7 ± 3.6	52.6 ± 4.0	0.292	95.2 ± 0.7	99.6 ± 0.8	0.208	123
Cysteine	4.9 ± 0.5	5.0 ± 0.5	0.664	9.2 ± 0.1	9.5 ± 0.1	0.510	12
Glutamic acid^d^	70.8 ± 5.8	74.0 ± 6.8	0.278	130.3 ± 1.1	140.2 ± 1.3	0.207	170
Glycine	47.1 ± 4.7	50.2 ± 5.8	0.217	88.4 ± 0.9	95.1 ± 1.1	0.170	116
Histidine	15.3 ± 1.5	15.3 ± 1.6	0.959	28.8 ± 0.3	28.9 ± 0.3	0.922	36
Isoleucine	22.5 ± 1.8	23.1 ± 2.1	0.518	42.3 ± 0.3	43.7 ± 0.4	0.403	54
Leucine	38.1 ± 2.9	39.6 ± 3.3	0.327	71.7 ± 0.6	75.0 ± 0.6	0.242	92
Lysine	41.6 ± 3.1	42.7 ± 4.3	0.514	78.1 ± 0.6	80.9 ± 0.8	0.407	100
Methionine	12.0 ± 1.1	12.4 ± 1.3	0.525	22.6 ± 0.2	23.4 ± 0.3	0.414	29
Phenylalanine	22.6 ± 1.7	23.3 ± 2.1	0.419	42.5 ± 0.3	44.1 ± 0.4	0.328	55
Proline	29.8 ± 2.6	31.1 ± 2.8	0.325	50.6 ± 0.5	58.8 ± 0.5	0.250	69
Serine	26.8 ± 2.5	26.7 ± 2.1	0.981	50.3 ± 0.5	50.6 ± 0.4	0.874	64
Threonine	22.6 ± 1.8	23.1 ± 2.0	0.592	42.5 ± 0.3	43.7 ± 0.4	0.467	54
Valine	23.2 ± 1.8	24.0 ± 2.0	0.391	43.7 ± 0.4	45.5 ± 0.4	0.296	56

**Notes.**

a Standard deviation.

b Body amino acid pattern (average of females and males) in grams per 100 g of lysine.

c During sample preparation, acid hydrolysis converted asparagine into aspartic acid.

d During sample preparation, acid hydrolysis converted glutamine into glutamic acid.

* *p* < 0.05.

**Table 4:** Mean+SD^a^ fatty acid composition of whole adult African clawed frogs (*Xenopus laevis*).

Fatty acid	Females *n* = 9	Males *n* = 10	*P*	Females *n* = 9	Males *n* = 10	*P*
	(g/kg Dry matter)			(g/kg Crude fat)		
Saturated fatty acids						
C10:0	0.04 ± 0.01	0.03 ± 0.0009	0.026^*^	0.20 ± 0.005	0.17 ± 0.005	0.185
C12:0	0.020 ± 0.06	0.13 ± 0.04	0.010^*^	1.00 ± 0.03	0.70 ± 0.02	0.055
C14:0	9.0 ± 3.1	5.8 ± 2.3	0.021^*^	44.6 ± 1.5	33.7 ± 1.3	0.092
C15:0	1.4 ± 0.3	1.0 ± 0.3	0.002^*^	7.0 ± 0.1	5.6 ± 0.2	0.032^*^
C16:0	45.9 ± 8.9	32.2 ± 10.2	0.007^*^	228.3 ± 4.4	185.9 ± 5.9	0.079
C17:0	1.1 ± 0.2	0.7 ± 0.2	0.002^*^	5.3 ± 0.1	4.3 ± 0.1	0.032^*^
C18:0	7.2 ± 1.5	5.8 ± 1.3	0.062	30.6 ± 0.7	33.6 ± 0.7	0.517
C20:0	0.6 ± 0.2	0.4 ± 0.2	0.076	3.0 ± 0.1	2.4 ± 0.1	0.268
C22:0	0.11 ± 0.04	0.09 ± 0.02	0.410	0.50 ± 0.02	0.50 ± 0.01	0.999
C24:0	0.08 ± 0.03	0.08 ± 0.02	0.768	0.40 ± 0.02	0.50 ± 0.009	0.490
Iso C15:0	0.4 ± 0.1	0.3 ± 0.1	0.052	2.0 ± 0.07	1.7 ± 0.06	0.215
Anteiso C15:0	0.17 ± 0.06	0.15 ± 0.07	0.217	0.8 ± 0.03	0.9 ± 0.04	0.614
Iso C16:0	0.20 ± 0.07	0.15 ± 0.06	0.050	1.0 ± 0.04	0.9 ± 0.04	0.202
Iso C17:0	0.8 ± 0.3	0.5 ± 0.2	0.048^*^	3.8 ± 0.2	3.0 ± 0.09	0.151
Total	67.0 ± 12.6	47.3 ± 14.1	0.009^*^	328.5 ± 6.8	273.9 ± 8.2	0.090
Mono unsaturated fatty acids						
C14:1	0.25 ± 0.06	0.17 ± 0.08	0.024^*^	1.2 ± 0.03	1.0 ± 0.04	0.132
C16:1 n-7	1.3 ± 0.2	0.9 ± 0.3	0.001^*^	5.9 ± 0.2	4.5 ± 0.2	0.022^*^
C16:1	25.1 ± 6.3	17.0 ± 8.6	0.034^*^	124.9 ± 3.1	98.5 ± 5.0	0.159
C17:1	0.8 ± 0.2	0.5 ± 0.1	0.005^*^	4.0 ± 0.1	3.0 ± 0.08	0.035^*^
Trans C18:1	1.5 ± 0.3	1.1 ± 0.4	0.089	7.3 ± 0.2	5.9 ± 0.3	0.457
C18:1 n-9	43.9 ± 7.9	33.2 ± 11.1	0.027^*^	218.5 ± 3.9	192.2 ± 6.4	0.236
C18:1 n-11	6.7 ± 1.1	4.9 ± 1.4	0.004^*^	33.6 ± 0.6	28.3 ± 0.8	0.065
C20:1	2.3 ± 0.7	1.7 ± 0.7	0.061	11.4 ± 0.3	9.9 ± 0.4	0.269
C22:1	0.2 ± 0.06	0.2 ± 0.07	0.143	1.0 ± 0.03	1.0 ± 0.04	0.505
C24:1	0.04 ± 0.005	0.03 ± 0.01	0.466	0.19 ± 0.003	0.2 ± 0.007	0.674
Total	82.1 ± 15.7	59.7 ± 22.3	0.022^*^	408.0 ± 7.8	345.0 ± 1.32	0.170
*n* − 6 Poly unsaturated fatty acids						
C18:2	5.7 ± 3.2	5.8 ± 3.7	0.965	28.2 ± 1.6	33.5 ± 2.1	0.669
C18:3	0.11 ± 0.03	0.08 ± 0.03	0.094	0.5 ± 0.02	0.5 ± 0.02	0.408
C20:2	0.8 ± 0.2	0.72 ± 0.02	0.337	3.8 ± 0.08	4.2 ± 0.01	0.760
C20:3	0.08 ± 0.07	0.09 ± 0.08	0.850	0.4 ± 0.04	0.5 ± 0.04	0.629
C20:4	0.23 ± 0.02	0.28 ± 0.03	0.697	1.1 ± 0.02	1.6 ± 0.02	0.517
C22:4	0.02 ± 0.01	0.02 ± 0.004	0.816	0.08 ± 0.009	0.10 ± 0.002	0.698
C22:5	0.02.0 ± 0.005	0.04 ± 0.01	0.661	0.10 ± 0.002	0.2 ± 0.006	0.606
Total	6.9 ± 3.5	7.0 ± 4.2	0.960	34.2 ± 1.7	40.6 ± 2.4	0.646
*n* − 3 Poly unsaturated fatty acids						
C18:3	0.7 ± 0.4	0.7 ± 0.6	0.848	3.5 ± 0.2	3.9 ± 0.3	0.835
C20:3	0.06 ± 0.02	0.05 ± 0.02	0.260	0.3 ± 0.01	0.3 ± 0.01	0.681
C20:4	0.09 ± 0.02	0.1 ± 0.03	0.839	0.5 ± 0.01	0.7 ± 0.02	0.740
C20:5	0.6 ± 0.1	0.9 ± 0.2	0.698	3.0 ± 0.01	5.1 ± 0.1	0.611
C22:5	0.2 ± 0.03	0.3 ± 0.6	0.655	0.9 ± 0.02	1.7 ± 0.04	0.577
C22:6	0.6 ± 0.09	1.0 ± 0.2	0.632	2.8 ± 0.05	5.6 ± 0.1	0.561
Total	2.3 ± 0.3	3.1 ± 0.6	0.778	11.0 ± 0.1	17.3 ± 0.3	0.645

**Notes.**

a Standard deviation.

* *p* < 0.05.

### Comparison with *Rana esculenta* and fish species

Comparing the proximate analysis and minerals with fish species, frogs generally showed a similar body composition profile, with fairly high protein and fat content. Among all species, *E. encrasicholus* was exceptional with higher water and fat, as well as lower ash. With respect to minerals, *X. laevis* contained more calcium and phosphorus than the other species, although *O. niloticus*’s calcium and phosphorus values agreed more with *X. laevis* than others (Table 5).

**Table 5:** Macronutrient and mineral profiles (dry matter basis) of adult whole African clawed frogs (*Xenopus laevis*),** compared to edible frogs (*Rana esculenta*) and several fish species.

Component	*Xenopus laevis^1^*	Edible Frog^a^	Black Sea Anchovy^b^	Commersons Anchovy^c^	Cod^d^	Tilapia^e^
Proximate analysis (%)						
Dry matter^2^	21.1	20.4	31.8	23.0	20.6	25.9
Crude protein	53.0	68.6	50.5	73.6	75.5	64.8
Crude fat	18.7	17.0	40.2	8.6	12.1	12.7
Ash	14.4	13.2	8.1	17.5	11.8	13.9
Macro minerals (g/kg)	****	****	****	****	****	****
Calcium	53.9	23.4	22.6	40.4	-^3^	43.0
Phosphorus	31.4	19.0	20.0	–	–	24.4
Magnesium	1.5	–	1.4	–	–	1.6
Potassium	8.5	9.8	8.3	8.2	–	14.1
Sodium	6.1	–	3.4	17.4	–	4.5
Micro minerals (mg/kg)	****	****	****	****	****	****
Copper	9.7	–	2.2	8.7	–	2.1
Iron	227.4	–	234.0	–	–	90.6
Manganese	14.2	–	22.1	13.0	–	3.2
Selenium	1.1	–	–	–	–	–
Zinc	106.6	296.4	130.1	43.4	–	115.5

**Notes.**

1 Average of females and males in the present study.

2 As is basis.

3 - NA: not available.

a *Rana esculenta* (farmed)*,* combined data of Tokur, Gürbüz & Özyurt (2008) and Oduntan et al. (2012).

b *Engraulis encrasicholus* (wild), Gencbay & Turhan (2016).

c *Stolephorus commersonii* (wild)*,* Sankar et al. (2013).

d *Gadus morhua* (wild), Shahidi et al. (1991).

e *Oreochromis niloticus* (farmed)*,* combined data of El-Saidy & Gaber (1998), Takeuchi et al. (2002) and Larbi-Ayisi, Zhao & Rupia (2017).

In relation to amino acids, the dominant amino acid in all the species was glutamate+glutamine, followed by aspartate+asparagine, glycine (except *O. niloticus*) and lysine, meanwhile methionine was lowest only in *X. laevis* and *S. commersonii*. Proline showed a substantial numerical difference between frogs and fishes (Table 6). As for fatty acid profiles, *E. encrasicholus* presented the highest fat and *n* − 3 PUFA content, whereas frogs presented the higher total MUFA and lower *n* − 3 PUFA concentrations. Despite *G. morhua* and *O. niloticus* presenting low fat and high MUFA concentrations, the latter species nonetheless displayed fat with a high SFA proportion (Table 7).

**Table 6:** Amino acid profiles of adult whole African clawed frogs (*Xenopus laevis*)** and comparison to edible frogs (*Rana esculenta*) and several fish species (% of total determined amino acids).

Amino acid (%)	*Xenopus laevis^1^*	Edible Frog^a^	Black Sea Anchovy^b^	CommersonsAnchovy^c^	Cod^d^	Tilapia^e^
Alanine	7.0	6.7	10.6	9.1	6.4	6.8
Arginine	5.9	7.0	6.8	0.6	6.9	6.7
Aspartic acid	10.3	7.8	14.1	12.0	10.6	12.7
Cysteine	1.0	-^2^	–	0.5	1.0	0.1
Glutamic acid	14.4	12.7	14.9	15.1	15.1	14.3
Glycine	9.8	10.7	8.5	11.3	9.8	4.2
Histidine	3.0	2.9	2.9	3.6	2.6	3.0
Isoleucine	4.6	4.1	3.4	5.1	4.3	6.3
Leucine	7.8	7.3	5.6	8.2	7.9	8.4
Lysine	8.5	6.5	9.5	8.9	8.6	9.3
Methionine	2.4	5.6	3.3	1.5	3.1	3.3
Phenylalanine	4.6	4.5	3.6	3.8	3.9	4.8
Proline	6.1	9.3	3.3	1.5	5.0	3.9
Serine	5.3	5.9	4.6	6.1	5.2	4.9
Threonine	4.6	4.2	4.5	5.6	4.7	5.3
Valine	4.7	4.8	4.4	7.1	4.9	6.0

**Notes.**

1 Average of females and males in the present study.

2 NA: not available.

a *Rana esculenta* (farmed)*,* combined data of Tokur, Gürbüz & Özyurt (2008) and Oduntan et al. (2012).

b *Engraulis encrasicholus* (wild), Gencbay & Turhan (2016).

c *Stolephorus commersonii* (wild)*,* Sankar et al. (2013).

d *Gadus morhua* (wild), Shahidi et al. (1991).

e *Oreochromis niloticus* (farmed)*,* combined data of El-Saidy & Gaber (1998), Takeuchi et al. (2002) and Larbi-Ayisi, Zhao & Rupia (2017).

**Table 7:** Fatty acid profile of whole adult African clawed frogs (*Xenopus laevis*)*,* with comparison to edible frogs (*Rana esculenta*) and several fish species (% of total determined fatty acids).

Fatty acid (%)	*Xenopus laevis^1^*	Edible Frog^a^	Black Sea Anchovy^b^	Commersons Anchovy^c^	Cod^d^	Tilapia^e^
Saturated fatty acids						
C10:0	0.02	–^2^	–	–	–	–
C12:0	0.1	–	0.09	0.63	–	–
C14:0	9.5	2.90	7.20	4.76	2.85	4.61
C15:0	0.8	–	1.24	1.78	–	–
C16:0	25.7	19.30	20.99	30.34	11.30	29.52
C17:0	0.6	–	1.84	1.35	–	–
C18:0	4.5	4.50	4.86	10.87	3.15	9.16
C20:0	0.3	–	1.20	–	0.30	2.96
C22:0	0.07	–	0.31	–	–	1.65
C24:0	0.06	–	0.65	–	0.70	–
Iso C16:0	0.1	–	–	–	–	–
Iso C15:0	0.2	–	–	–	–	–
Anteiso C15:0	0.1	–	–	–	–	–
Iso C17:0	0.4	–	–	–	–	–
Total	41.7	26.70	38.67	57.28	18.30	47.90
Mono unsaturated fatty acids						
C14:1	0.1	–	0.04	–	0.20	–
C16:1 n-7	0.7	10.80	–	5.26	–	–
C16:1	13.4	–	6.30	–	9.85	6.27
C17:1	0.4	–	0.26	0.11	–	–
Trans C18:1	0.9	–	0.11	–	–	–
C18:1 n-9	25.5	26.00	14.18	10.16	20.50	28.98
C18:1 n-11	3.9	–	–	–	–	–
C20:1	1.3	5.70	1.06	0.27	10.40	–
C22:1	0.1	–	0.47	0.56	6.45	–
C24:1	0.03	–	0.90	–	1.60	–
Total	47.1	42.50	23.32	17.19	49.00	36.06
*n* − 6 Poly unsaturated fatty acids						
C18:2	3.9	16.70	2.24	1.24	2.30	3.28
C18:3	0.07	0.30	0.15	–	–	–
C20:2	0.5	–	2.15	0.50	0.30	–
C20:3	0.06	–	0.08	0.54	–	–
C20:4	0.2	–	0.81	2.03	0.45	1.44
C22:4	0.02	–	–	–	0.50	–
C22:5	0.03	–	–	–	–	–
Total	4.8	17.00	6.02	4.31	3.55	4.72
*n* − 3 Poly unsaturated fatty acids						
C18:3	0.4	0.20	1.56	2.17	0.35	1.05
C20:3	0.4	0.40	0.14	–	–	–
C20:4	0.03	–	–	–	–	–
C20:5	0.2	1.80	10.24	2.87	8.30	–
C22:5	0.06	–	–	–	1.30	0.56
C22:6	0.2	0.90	20.05	13.83	12.35	1.61
Total	1.3	3.30	31.99	18.87	22.30	11.32
Others^3^	5.1	10.5	–	2.3	6.8	-

**Notes.**

1 Average of female and male of the present study.

2 –NA: not available.

3 Other not specified fatty acids not analysed.

a *Rana esculenta* (farmed)*,* combined data of Tokur, Gürbüz & Özyurt (2008) and Oduntan et al. (2012).

b *Engraulis encrasicholus* (wild), Gencbay & Turhan (2016).

c *Stolephorus commersonii* (wild)*,* Sankar et al. (2013).

d *Gadus morhua* (wild), Shahidi et al. (1991).

e *Oreochromis niloticus* (farmed)*,* combined data of El-Saidy & Gaber (1998), Takeuchi et al. (2002) and Larbi-Ayisi, Zhao & Rupia (2017).

### Associations between parameters in the combined *X. laevis* data

**Figure 2:**
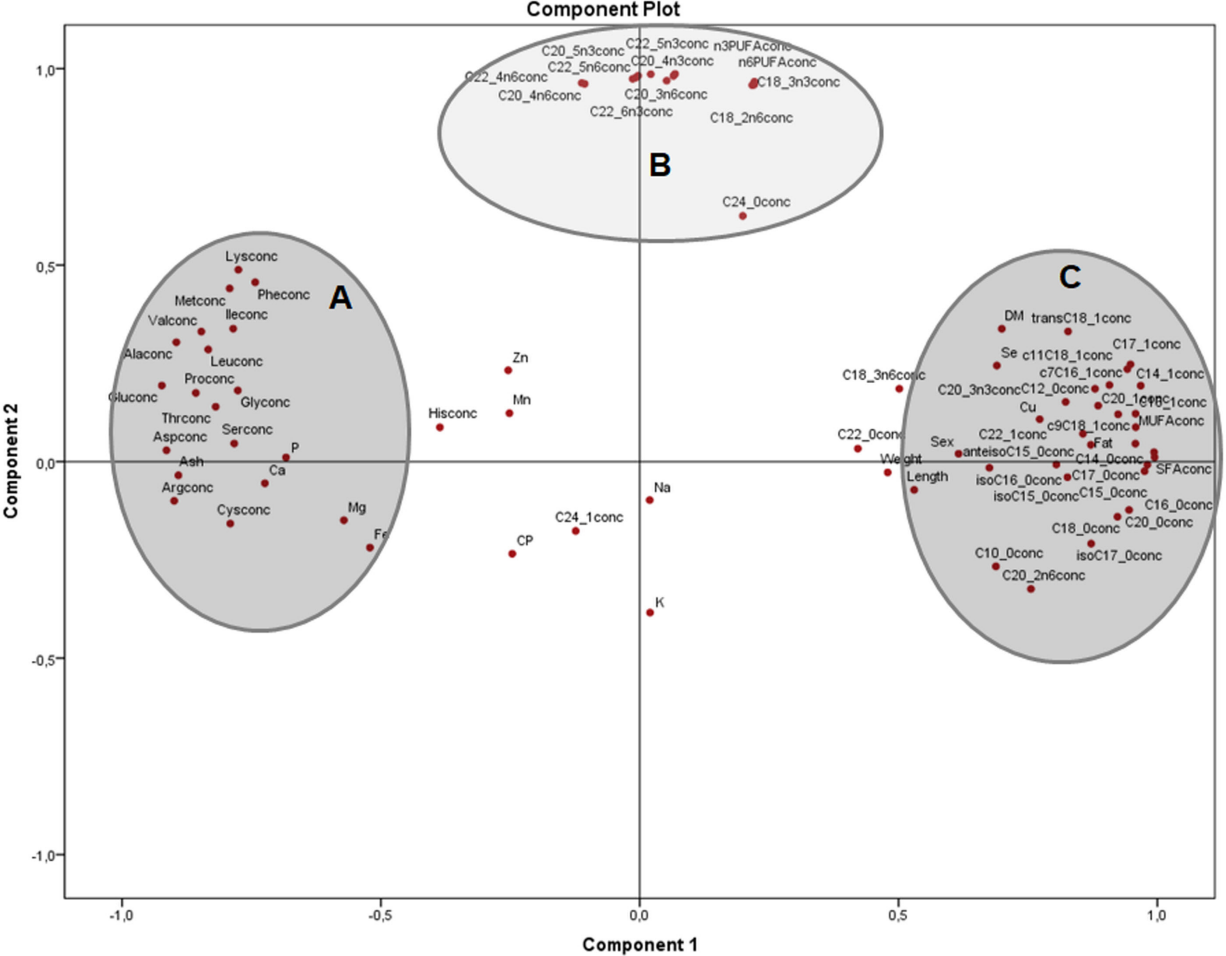
Principal components analysis (PCA) plot of morphometric measurements and concentration of nutrients (on dry matter basis) of the combined (male plus female) whole *X. laevis* body composition data. Conc: concentration. Group A closely clustered in loadings: Amino acids, Ca: calcium, P: phosphorus, Mg: magnesium, Fe: iron. Group B strong relationships: polyunsaturated fatty acids. Group C closely clustered in loadings: Saturated and monounsaturated fatty acids, weight and length, among others.

Analysis of nutrients in terms of concentrations (Fig. 2) clearly shows the main groups of macronutrients in the frogs body, with two clusters on component 1, one with amino acids and minerals (A) and the other with morphometric measurements and most of SFA and MUFA (C). From component 2, independent of amino acids, minerals and saturated fatty acids, there is a group with many *n* − 3 and *n* − 6 PUFA (B). In this plot, component 1 explained 50% and component 2, 19.2% of the variation, respectively

**Figure 3:**
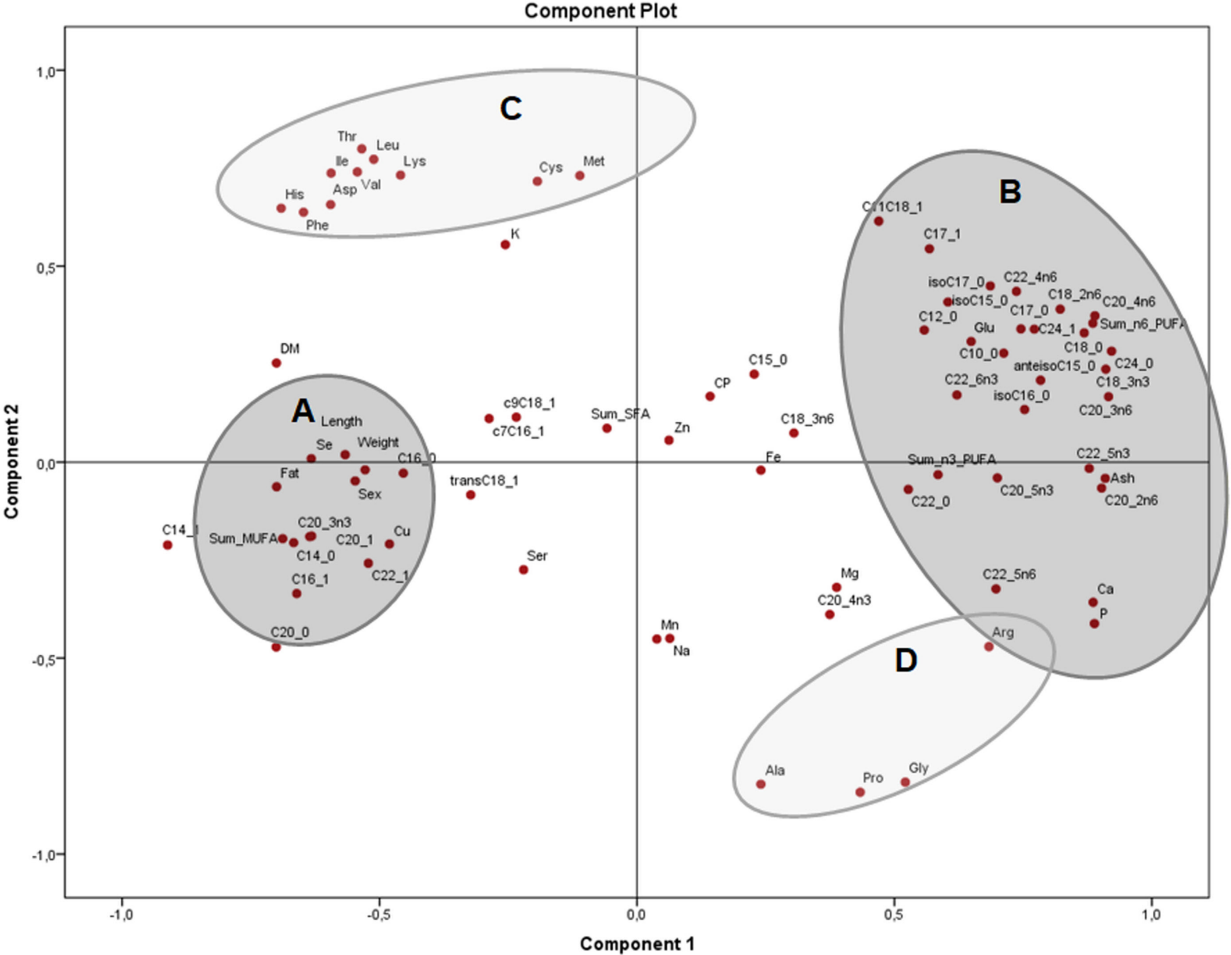
Principal components analysis (PCA) plot of morphometric measurements and nutrients (expressed as percentages of the sum of total) of the combined (male plus female) whole *X. laevis* body composition data. Group A closely clustered in loadings: Se: selenium, Cu: copper, SumMUFA: sum of monounsaturated fatty acids, Group B closely clustered in loadings: Glu: glutamic acid, Ca: calcium, P: phosphorus, SumPUFA: sum of polyunsaturated fatty acids. Group C (essential amino acids): Leu: leucine, Ile: isoleucine, Phe: phenylalanine, His: histidine, Thr: threonine, Lys: lysine, Asp: aspartic acid, Val: valine, Cys: cysteine, Met: methionine. Group D (non-essential amino acids): Ala: alanine, Pro: proline, Gly: glycine, Arg: arginine, among others.

The principal components plot displays the relationships of morphometric measurements and all nutrients of *X. laevis* bodies in terms of relative data (percentages) (Fig. 3) explaining 54.5% of the variation (37.9% and 16.6% from components 1 and 2, respectively). Along the component 1 axis, group A showed positive relationships between weight and length with selenium, copper and crude fat, as well as several monounsaturated fatty acids, while group B presented positive associations with glutamic acid and different fatty acids, ash, calcium, phosphorus and arginine. Group A also demonstrated how sex is strongly related to morphometric measurements. Likewise, the inverse associations between Groups A and B comprise the clear contrast between the fatty acid profiles (MUFA and PUFA), fat and macro minerals (calcium and phosphorus) and morphometric measurements (with a wide profile of individual fatty acids and macro minerals), as well as in some amino acids from groups C and D. Additionally, component 2 conspicuously had two strongly contrasting groups of amino acids (Fig. 3). This component exhibited two groups of these nutrients, separated into essential (Group C) and non-essential (Group D) amino acids, also showing inverse associations between these constituents. Weight, length and fatty acid values did have a minor contribution to either positive or negative associations in this particular assessment.

**Figure 4:**
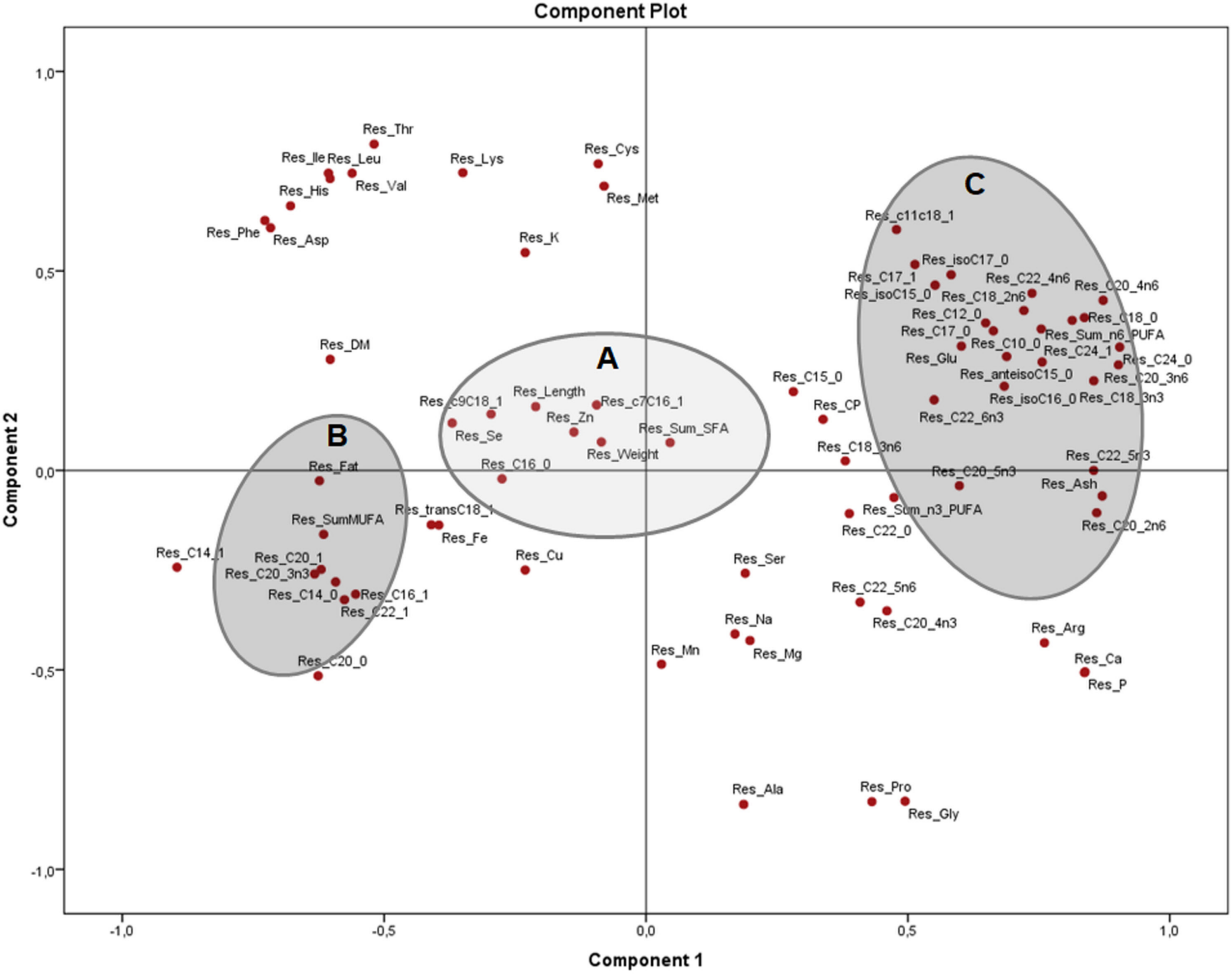
Principal components analysis (PCA) plot with the residuals (corrected by sex) of morphometric measurements and nutrients (expressed as percentages of the sum of total) of the combined (male plus female) whole *X. laevis* body composition data. Res: residuals. Group A closely clustered in loadings: Zn: zinc, Se: selenium, SumSFA: sum of saturated fatty acids. Group B closely clustered in loadings: SumMUFA: sum of monounsaturated fatty acids. Group C closely clustered in loadings: Glu: glutamine, SumPUFA: sum of polyunsaturated fatty acids, among others.

The PCA of the residuals after accounting for sex (Fig. 4) showed how variables from group B (fat, total MUFA) and group C (glutamic acid, ash and several PUFA) remained grouped and inversely related as seen in Fig. 2, while group A (weight, length, selenium, the sum of SFA and several MUFA) are clustered in the centre with no significant association to B and C. This indicates that these associations are independent of sex and morphometric measurements. Both components explained 51.4% of the variation (33.4 and 18.0% from components 1 and 2, respectively).

## Discussion

### Differences between sexes

Female frogs were 51.3% heavier and 25.6% longer than males. This pattern of sexual size dimorphism occurs in approximately 90% of anuran species, and is explained mainly by fecundity selection and high survival rates compared to males, following the patterns of many other lineages of poikilothermic vertebrates in which females are the larger sex (Shine, 1979; Kupfer, 2007). Likewise, results showed also concomitant differences in their body composition. Schulte-Hosedde, Millar & Hickling (2001) reported differences between sexes in bushy-tailed wood rats (*Neotoma cinerea*), deer mice (*Peromyscus maniculatus*) and red-backed voles (*Clethrionomys gapperi*), with males having more body water and a leaner dry mass composed primarily of ash. Similarly, the proximate composition differences reported between sexes in the fresh water catfish *Wallagu atto* (Yousaf, Salam & Naeem, 2011), agreed with the findings from this study in *X. laevis*. Water is more closely related to protein than fat; although both parameters showed no differences between sexes, females contained numerically more fat than males, which could influence the lean tissue proportion (muscle and ash).

Females normally carry more fat than males to produce large and energy-rich macrogametes, as well as to facilitate successful production of offspring, while males could require a more robust skeleton to provide them advantages in mate searching and combat, where well-endowed individuals would pair successfully with the females (Andersson, 1994; Schulte-Hosedde, Millar & Hickling, 2001). These characteristics also might impact sex-specific dietary nutritional requirements as well as their own chemical composition, with ultimate goals of optimizing nutrient performance to best meet needs at different physiological stages. In production animals, diets are designed to maximize outputs and reduce losses, taking into account the dynamics of metabolism of the animal. Sex-specific differences in some nutrient requirements and utilisation during growth, reproduction, lactation and laying periods, for example, are recognised in livestock (McNamara & Phillips, 2000; McDonald et al., 2011) but have not previously been considered for amphibians.

Since the proportion of the skeleton in female bodies is lower than in males, associated nutrients such as total minerals (ash), calcium and phosphorus (possibly even magnesium) might also be expected to be lower. As a proportion of total minerals, however, females showed higher concentrations of selenium and a trend towards higher copper levels. In general, selenium and copper are related to soft tissues (muscles, kidneys, liver and brain). Selenium is a component of selenocysteine and selenomethionine, which are both sources of selenium suitable for the synthesis of selenoproteins (SePs), and also linked with glutathione metabolism. Copper aids the incorporation of iron into haemoglobin, particularly concentrated in the liver (McDonald et al., 2011). Several studies have indicated sex differences in the metabolism of various trace minerals. Yeh et al. (1998) demonstrated that female rats (*Rattus norvegicus domesticus*) have a higher content of selenoprotein W in muscle and skin, higher glutathione peroxidase activity in the brain, liver, stomach and ovaries and higher selenium in the liver, while Smith, Cha & Kimura (1995) suggested that changes in both selenium and glutathione in females might be related to hormonal fluctuations and the stage of the rats reproductive cycle. Likewise, copper concentrations showed important sex differences in brain, liver and kidney of rats, being higher in females than males (Uchino, Tsusuki & Inoue, 1990).

The close match in amino acid profiles between the female and male frogs in this study indicates that animals have a relatively stable amino acid pattern, which also has been documented in both sexes of whole lumpfish, *Cyclopterus lumpus* (Njaa & Utne, 1982), as well as in the edible portion of the common carp *Cyprinus carpio* (Buchtová et al., 2009). Fatty acid profiles varied between sexes, with females containing greater proportions of SFA and MUFA, and males numerically more PUFA, particularly when expressed on a total body (rather than fat tissue) basis. Similar findings have been reported in the cultured brook trout *Salvelinus fontinalis*, Black Sea trout *Salmo tutta labrax* (Şahin et al., 2011), tilapia *O. niloticus* (Abelti, 2017) and the sea lamprey *Petromyzon marinus* (Happel, Rinchard & Czesny, 2017). These results indicate that both sexes may possess different mechanisms for (or different requirements/pressures on) metabolism of certain fatty acids. Such observations warrant further investigation concerning sex differences in fatty acid profiles, as well as enzymatic reactions and specific pathways related to nutrient requirements in *X. laevis*.

### Comparison with fish and *R. esculenta*

In general, the macronutrient profile of *X. laevis* was very similar to the species compared, except *E. encrasicholus*. Likewise, calcium and phosphorus values were higher in *X. laevis* than other species, indicating that both groups possess particular calcium mechanisms compared to endothermic species (Srivastav et al., 2000; Takeuchi et al., 2002). Despite the general similitude with most amino acids, *X. laevis* showed some peculiarities in the case of methionine, cysteine and proline. The low level of methionine in the frog may be linked to different interactions with other amino acids, in this case, cysteine. Although speculative, methionine might be rapidly transsulphurated to cystathione and cysteine, and subsequently to glutathione and/or taurine to function as antioxidants, although more evidence is necessary to confirm this. In addition, proline was higher in *X. laevis* than in all fish compared. This structural amino acid, together with hydroxyproline, comprises the major components of collagen and cartilage present in the skin and connective tissues (Michal & Schomburg, 2012), hence these constituents may have a higher distribution in the frogs body compared to fish.

The differences found in the fatty acid profiles of *X. laevis* compared with fish species provide a first insight into anuran fat characterisation. The high MUFA and *n* − 6 PUFA found in *X. laevis* suggests that the frog does not behave like fish but rather like terrestrial animals, often showing lower *n* − 3 PUFA, and tending to accumulate instead saturated fats (Lladó, Pons & Palou, 1997; Fujiwara et al., 2015). Certainly, those contrasts may also be impacted by diet and the origin of the animals (wild versus cultivated). While dietary fatty acids have been demonstrated to modify body tissue profiles in several terrestrial species (Crespo & Esteve-Garcia, 2001; Plantinga & Beynen, 2003), *R. esculenta* showed a similar profile to *X. laevis* in this study, indicating some possible particularities related to this taxonomic group. In this regard, there is also evidence of a similar fatty acid profile in the marsh frog (*Rana ridibunda*), both from the wild as well as captivity (Cagiltay et al., 2014). These fatty acid metabolic pathways have neither been well characterised, nor been the subject of direct comparisons made between aquatic and terrestrial species, for anurans.

The results obtained from the chemical analysis indicate that frogs likely have similarities that overlap with both marine and fresh water fishes. Considering the group’s broad diversity, generalizations should be made with caution; distinctions among species, incorporating ecological and physiological adaptations must be considered in order to understand the complexity of nutrient dynamics and mechanisms in anurans.

### Associations between parameters in the combined *X. laevis* data

The PCA tests detected not only engaging clustering among nutrients, sex and body measurements, but also showed that interpretation of data does depend on the units utilised for the analysis. In the first instance, the analysis in terms of nutrient concentrations on a dry matter basis (Fig. 2) provides information regarding how the nutrients are distributed per amount of dry matter, exhibiting a profile of differentiation depending on the tissues (muscle, skeleton, fat deposits). However, that profile does not indicate the types and relationships of nutrients in those tissues, which can be observed when evaluated in terms of relative values (Fig. 3), because the composition of the dry matter is dependent on the relative proportions of nutrients (McDonald et al., 2011).

When amino acid and fatty acid proportions were included in the PCA (Fig. 3) a close relationship between body weight and sex as a result of allometric effects, with several chemical components distributed differently in the body (McDonald et al., 2011), was observed. Results from component 1 variables (Group A) demonstrated direct associations between weight and length with fat content, as well as body selenium and copper. In this regard, most of the copper is considered active or in transit within the body, acting as a cofactor in several enzymes and electron transport proteins involved in energy or antioxidant metabolism (Linder & Hazegh-Azam, 1996). Likewise, selenium incorporated into selenoproteins plays a critical role in optimum protein function and is distributed throughout the animal’s body (Shini, Sultan & Bryden, 2015).

The strong positive association of morphometric measurements and MUFA in *X. laevis* suggests that MUFA are important contributors to size and weight in these frogs. However, the PCA of the residuals (Fig. 4) showed that clusters of fat and MUFA do not seem to depend exclusively on sex and body measurements, meaning that other, yet unknown effects were dominating these associations. The overall body fatty acid composition might be strongly related to dietary abundance (Tokur, Gürbüz & Özyurt, 2008). In frogs, the majority of the lipids are stored in the fat bodies, with a reported 41% corresponding to saturated and 59% to unsaturated fatty acids (Fitzpatrick, 1976). High levels of MUFA have also been mainly associated with adipose tissue in adult bulls (*Bos taurus* and *B. indicus;* Smith et al., 2013) and rats (Fujiwara et al., 2015), as well as in skin, fins and tails of different species of fish (Sahari et al., 2013). Clearly, further detailed information on tissue analysis and effects of the diet are required to better understand relationships to frog body composition.

PUFA, as well as ash, calcium and phosphorus, were negatively associated with frog weight and length, indicating that the profile of fatty acids can vary depending on how “fat” or “fit” the animal is (recalling that fitness is also related to the skeletal composition in terms of calcium and phosphorus content). Additionally, the residuals showed that such associations could be derived from other causes, which is not yet fully understood in the case of anurans.

Component 1 (Group B) also showed a strong positive association of glutamate with several fatty acids as well as with arginine (Fig. 3). Glutamate plays a key central role in amino acid interconversions, and comprises a high proportion of the body pool of amino acids in the free form, but also incorporated into proteins. Glutamate also can be condensed with acetyl-CoA to yield N-acetylglutamate, the initial compound for ornithine synthesis, which in turn can be converted into arginine (Michal & Schomburg, 2012; Watford, 2015). On the other hand, fatty acid synthesis from glutamate has been reported in animals, taking place in the cytosol through the backward pathway of the Krebs cycle, where glutamate is converted to 2-oxo-glutarate and thereupon to citrate, which is split to yield acetyl-CoA needed for fatty acid biosynthesis (Madsen, Abraham & Chaikoff, 1964; Michal & Schomburg, 2012). Although speculative, given the relationships shown by *X. laevis* in the current study, it could be assumed that frogs also share these metabolic pathways, though more evidence remains to be addressed in controlled studies.

Remarkably, independent of sex, results of associations from component 2 of the PCA (Fig. 3) indicated that the small amino acids such as proline, glycine, alanine and also arginine (Group D), seem to separate from the others (Group C). Proteins of connective tissues like collagen and elastin are rich in proline, alanine and glycine, while arginine plays an important role in the urea cycle (McDonald et al., 2011; Michal & Schomburg, 2012). Amino acids from Group C (Fig. 3) are mostly found in muscle and organ tissues from many species, including frogs, with muscles containing high lysine, and heart, liver, kidney and brain more phenylalanine (Beach, Munks & Robinson, 1943; Cagiltay et al., 2014). Interestingly, arginine being grouped with the non-essential amino acids suggests that obligate, frequently feeding carnivores (for which arginine is a dietary essential; Bosch, Hagen-Plantinga & Hendriks (2015) may not be the most suitable physiologic model for amphibians, although this goes beyond the scope of the current study. These integrated nutrient profiles, complemented with both absolute and relative data, provide valuable observations regarding nutrient dynamics in the frog body, which ultimately might be indicators of frog nutrient requirements.

## Conclusion

The frogs were high in protein and moderate in fat content, with sex-specific mineral and fatty acid differences, but similar amino acid patterns. Compared to a number of fish species, *X. laevis* showed differences in nutrient profile, especially in methionine, cysteine and proline, suggesting a high synthesis and accretion of proteins rich in these amino acids, and contrasting with other amino acids. On this captive diet, frogs tended to accumulate SFA and MUFA, a pattern also seen in several terrestrial species (compared with aquatic species) and some anurans from the wild. Sex and size could explain part of the associations found among body nutrients.

## Additional Information and Declarations

### Data Availability

The following information was supplied regarding data availability: Raw data is available at GitHub (https://github.com/Abreso/Whole-bodies-Xenopus).
